# Sorption of phenols and flavonoids on activated charcoal improves protein metabolism, antioxidant status, immunity, and intestinal morphology in broilers

**DOI:** 10.3389/fvets.2023.1327455

**Published:** 2024-01-11

**Authors:** Ying Zhang, Xiaoqi Fu, Lixue Wang, Xiangyue Guo, Bing Dong

**Affiliations:** ^1^State Key Laboratory of Animal Nutrition, College of Animal Science, China Agricultural University, Beijing, China; ^2^Institute for Hepatology National Clinical Research Center for Infectious Disease, Shenzhen Third People’s Hospital, Shenzhen, China; ^3^Plateau Animal Nutrition and Feed Laboratory, Tibet Agriculture and Animal Husbandry University, Nyingchi, China

**Keywords:** carbon, hematology, growth performance, scavenging activity, correlation analysis

## Abstract

Previous studies have revealed that activated charcoal sorption of Chinese herbal extracts is more effective than activated charcoal. The present study was designed to investigate whether phenols and flavonoids have an effect on nutrient metabolism, antioxidant activity, immunity, and intestinal morphology in broilers. Seven diets [basal diet (CON); CON supplemented with 450 mg/kg of activated charcoal (AC); CON supplemented with 250, 500, 750, 1,000, or 7,500 mg/kg of phenolic acids and flavonoids (PF) to AC (PFAC)]. PFAC was the complex of AC sorption of PF in the ratio of 9:1. These dietary treatments for broilers lasted for 42 days. Results showed that at d 21, all doses of PFAC altered serum levels of total protein, albumin, and creatinine compared to AC (*p* < 0.05). Both PFAC and AC altered HDL-, LDL-, and VLDL-cholesterol levels compared to CON (*p* < 0.05). PFAC at 500 mg/kg (450 mg/kg AC+ 50 mg/kg phenolic acids and flavonoids) increased serum IgA and IgM (*p* < 0.05), but AC at 450 mg/kg did not, compared to CON. At d 42, breast and thigh muscles of PFAC-treated broilers had higher free radical scavenging activities compared to CON (*p* < 0.05), but AC had no such effect. PFAC at 500 mg/kg increased villus height in the duodenum, jejunum, and ileum compared to CON (*p* < 0.05), but AC had no such response. PFAC at 500 mg/kg effectively improved protein and lipid metabolism, antioxidant status, and intestinal morphology, but AC had no such effect at a similar dose. Excessive PFAC (7,500 mg/kg) showed no significant side effects on broiler growth, liver damage, or hematology. These results suggest that phenols and flavonoids, in cooperation with activated charcoal, provide the majority of the functions of the herbal extract from multiple Chinese medicinal herbs.

## 1 Introduction

Commercial broilers endure multiple stressors during growth. Fast growth, associated with high metabolic rates and the high nutrient density of their feed, can easily cause local tissue hypoxia (1). Similarly, mycotoxins, mycotoxin metabolites, and pathogens in the feed can provoke excessive production of free radicals and tissue inflammation (2). Accumulated free radicals generated by oxidative stress can damage DNA, proteins, and cell membranes, which in turn can damage tissue structure and cause dysfunction of the tissues and organs. The indices of antioxidant enzymes, immune factors, and local inflammatory factors are indicators of multiorgan system deterioration (3, 4). Antibiotics have been used to mitigate the detrimental effects of oxidative stress on broiler productivity. However, concerns about the development of antibiotic resistance and residues in the environment led the European Union to ban the use of growth-promoting levels of antibiotics in animal production in 2006 (5). Subsequently, China instituted a similar ban in 2020 (6).

Charcoal is a carbon-rich material produced by the pyrolysis of biomass at temperatures between 300 and 1,000°C in a low-oxygen environment (7). Historically, charcoal has been used in China for the treatment of human and animal diarrhea (8, 9). Activated charcoal is produced in the presence of activation reagents to yield a highly porous structure containing alkali bonds. Because of this structure, activated charcoal can adsorb mycotoxins (10, 11) and most bacterial toxins (12), which can improve intestinal morphology (13), alleviate diarrhea (14, 15), and reduce intestinal inflammation (16). Consequently, numerous studies have reported the beneficial effects of activated charcoal as a feed additive in various species, including pigs and chickens (17–21). The effective dose of dietary activated charcoal for growth promotion ranges from 0.3 to 10% (22).

In addition to adsorption, activated charcoal possesses redox activity, which means it can accept, store, and mediate electrons to and from biochemical reactions (23). The electrical conductivity of activated charcoal makes it an electron mediator or an electron shuttle to facilitate electron transfer (24). Coating charcoal with liquids or organic nutrients can alter the internal surfaces of the porous charcoal by increasing its hydrophilicity, redox-active moieties, and additional mesoporosity (25, 26). This coating process can strengthen the water-holding capacity and ability to exchange nutrients in activated charcoal. Blending activated charcoal with wood vinegar or humic substances enhances the anti-bacterial activity of activated charcoal (27, 28).

One of our previous studies found that the sorption of Chinese herb extracts into activated charcoal promoted growth, reduced inflammation, improved antioxidant status, and modulated the intestinal microbiota (29–31). The effective dose of activated charcoal with Chinese herb extract was about one-tenth of the effective dose of activated charcoal without sorption (29–31). The explanation was the possible interaction between activated charcoal and the organic substances in Chinese herb extracts (28). The extracts of Chinese herbs contain several components, including phenols, flavonoids, and organic acids (28), and these may enhance the redox-buffering effect of activated charcoal. In another of our studies, phenols and flavonoids were shown to account for the majority of the detoxifying effects of herbal extracts (32). However, it is still unclear whether the sorption of phenolic acids and flavonoids into activated charcoal can promote growth *in vivo*. Therefore, in this study, we investigated the sorption of phenolic acids and flavonoids contained in herbal extracts into activated charcoal on growth performance, nutrient metabolism, antioxidant status, immune status, and intestinal morphology of broilers.

## 2 Materials and methods

All procedures used in this study were conducted in accordance with the Chinese Guidelines for Animal Welfare and approved by the Institutional Animal Care and Use Committee of China Agricultural University (AW52501102).

### 2.1 Preparation of activated charcoal sorption

The preparation of phenolic acids and flavonoids (PF) sorption to activated charcoal (PFAC) followed previously described methods (32), except that phenolic acids and flavonoids were products (PF product) from Xi’an Victory-Bio Ltd. Co. (Xi’an, China) (Lot no. DXE2013453), which were derived from methanolic extraction of *Portulaca oleracea L*. containing >80% of total phenolic acids and flavonoids [490 ± 4 mg/g total phenols (gallic acid equivalents) and 315 ± 3 mg/g total flavonoids (quercetin equivalents)]. Other components included 18 ± 1 mg/g total saponins (escin equivalents), 14 ± 1 mg/g total alkaloids (caffeine equivalents), and 14 ± 1 mg/g total carotenoids (β-carotene equivalents). Before the preparation of PFAC, the above parameters were measured in the laboratory to confirm their accuracy by using quantified spectrophotometry. Approximately 100 mg of the methanolic extract of *Portulaca oleracea L*. was dissolved in 25 mL of the respective solvents and quantified spectrophotometrically (Hitachi U-2001 spectrophotometer, Tokyo, Japan) to determine the antioxidant compounds: total phenolics content, total flavonoids content, total saponins content (33), total alkaloids content (34), and total carotenoids content (35). The phytoconstituents in both extracts were calculated using the respective standard curves, and the results were expressed as mg standard equivalents/g extract. All determinations were performed in six replicates. The ratio of PF products to activated charcoal was 1:9 (w/w) in the final PFAC, according to our previous study (32). Specifically, 250 mg/kg of PFAC contained 225 mg of AC and 25 mg of PF; 500 mg/kg of PFAC contained 450 mg of AC and 50 mg of PF; 750 mg of PFAC contained 675 mg of AC and 75 mg of PF, and so on.

### 2.2 Animals, diets, and experimental design

Male Arbor Acres (AA) broiler chicks were given *ad libitum* access to feed and water. Room temperature was maintained at 34°C–35°C during the first 7 d of the experiment, then gradually decreased by 2°C per week to 25°C–26°C, which was maintained thereafter. The birds received 24 h of continuous light throughout the experiment. Treatments included a corn-soybean meal basal diet formulated to meet the nutrient requirements of broilers (NRC, 1994) (Table 1). To prepare the treatment diets, PFAC was first mixed with a premix, which was then mixed with other feed ingredients. The diets were prepared in one batch for the subsequent feeding experiment. Samples of all experimental diets were collected and stored at −20°C prior to mycotoxin analysis using Ultra Performance Liquid Chromatography (36). Broilers were vaccinated against Newcastle disease on d 7 and against infectious bursal disease on d 14. The experiment was carried out at the National Feed Engineering Technology Research Center of the Ministry of Agriculture Feed Industry Center Animal Testing Base (Hebei, China).

**Table 1:** Composition and analyzed nutrient concentration of basal diets (%, dry matter basis).^a^

Item	Starter Phase (d 1–21)	Grower Phase (d 22–42)
Corn	59.85	60.80
Soybean meal	30.13	28.63
Fish meal	4.00	2.77
Soybean oil	2.75	4.54
Dicalcium phosphate	0.88	1.00
Limestone	1.45	1.38
98% DL-Methionine	0.14	0.08
Salt	0.30	0.30
Vitamin-mineral premix^a^	0.50	0.50
Total	100.00	100.00
Nutrient levels		
Digestible energy, kcal/kg	2222.00	3107.00
Crude protein	21.46	20.00
Calcium	0.99	0.95
Total phosphorus	0.68	0.67
Methionine	0.50	0.41
Lysine	1.13	1.04

a The premix provided the following per kilogram of the diet: vitamin B1, 300 mg; vitamin B2, 1,200 mg; vitamin B6, 600 mg; vitamin B12, 2.4 mg; niacin, 6,000 mg; pantothenic acid, 2,400 mg; folic acid, 60 mg; biotin, 2.4 mg; iron, 18 g; copper, 2 g; zinc, 15 g; manganese, 14 g; iodine, 120 mg; and selenium, 66 mg. Nutrient levels are calculated values.

Experiment 1 was designed to evaluate the effects of PFAC at different doses on lipid metabolism and biochemical indices in broilers. Male AA chicks (1 d old, initial body weight (42.8 ± 0.6 g)) were assigned randomly to one of six dietary treatments (*n* = 6 pens/treatment; *n* = 18 birds/pen): a corn-soybean meal basal diet supplemented with 0, 250, 500, 750, 1,000 mg/kg of PFAC or 450 mg/kg of activated charcoal (AC), respectively. Experiment 2 was conducted to determine the safety of excessive PFAC supplementation in broilers. Male AA chicks (1 d old, initial body weight (41.9 ± 0.6 g)) were assigned randomly to one of three treatments (*n* = 6 pens/treatment; *n* = 18 birds/pen) fed with a corn-soybean meal basal diet supplemented with 0, 750, or 7,500 mg/kg of PFAC, respectively. Each experiment lasted 42 d, which included a starter phase (1–21 d) and a growth phase (22–42 d). Body weights of the birds were recorded on d 1, d 21, and d 42 of the experiment after 12 h of feed but not water deprivation. Body weight gain (BWG) was calculated for the following periods: d 1 – d 21, d 22 – d 42, and d 1 – d 42. Feed intake (FI) was recorded for the same periods, and the ratio of gain to feed (G:F) was calculated after correcting for mortality.

### 2.3 Collection of blood and organ samples

On d 21 and d 42, one broiler closest to the average body weight of the birds in each pen was selected for blood sampling. Blood samples were collected from the jugular vein into vials containing EDTA. Serum was prepared at 4°C and stored at −20°C for further analysis. After blood sampling, one bird from each replication was euthanized by cervical dislocation, and three segments of the small intestine, duodenum, jejunum, and ileum, were collected and soaked in paraformaldehyde (4%) for histological analysis. Liver and kidneys were collected according to the procedures of Gasparotto et al. (37), packaged under vacuum conditions, and preserved at −80°C for further analysis. Breast and thigh muscles without skin from the left side of the body were collected, chilled in liquid nitrogen, and stored at −80°C for further analysis.

### 2.4 Assay of hematological parameters

Red blood cell (RBC) count, hemoglobin (HGB), hematocrit (HCT), mean corpuscular volume (MCV), mean corpuscular hemoglobin (MCH), mean corpuscular hemoglobin concentration (MCHC), platelet count (PLT), plateletcrit (PCT), platelet distribution width (PDW), and mean platelet volume (MPV) were determined for each sample of whole blood. Hematological parameters of the blood were determined using a hematology analyzer (Hematology Analyzer TEK8532, Shanghai, China).

### 2.5 Assay of serum biochemical parameters

The blood sample was prepared by centrifugation at 3,000 × g for 15 min at 4°C. The supernatant obtained was employed to analyze the following serum markers: glucose, total protein (TP), albumin (ALB), creatinine (CREA), urea, alkaline phosphatase (ALP), alanine aminotransferase (ALT), and aspartate aminotransferase (AST). The biochemical parameters were analyzed using an automated biochemistry analyzer (Hitachi 7,600, Japan).

### 2.6 Analysis of indices of inflammatory and immune factors, antioxidant status, and lipid metabolites

Concentrations of malondialdehyde (MDA), interleukin-1β (IL-1β), insulin-like growth factor 1 (IGF-1) in serum and intestine, and interferon-γ (IFN-γ), immunoglobulins (IgG, IgM, and IgA) in serum were determined using commercially available kits (Nanjing Jiancheng Bioengineering Institute, Nanjing, China). Intestinal secretory immunoglobulin (SIgA) concentrations were assayed using an Sn-69513-type immune counter (Shanghai Nuclear Annular Photoelectric Instrument Co., Ltd., Shanghai, China). Antioxidant indices in the serum, liver, kidney, and intestines, including catalase (CAT) and glutathione peroxidase (GSH-Px) activities, were measured using commercially available kits (Nanjing Jiancheng Bioengineering Institute, China) according to the manufacturer’s instructions. Serum total cholesterol, high-density lipoprotein cholesterol (HDLC), low-density lipoprotein cholesterol (LDLC), and very low-density lipoprotein cholesterol (VLDLC) were analyzed using an automated biochemistry analyzer (Hitachi 7,600, Japan).

### 2.7 Analysis of radical scavenging activity

Radical scavenging activities in breast and thigh muscles using 2,2-diphenyl-1-picrylhydrazyl (DPPH) and 2,2′-azino-bis(3)-ethylbenzthiazoline-6-sulfonic acid (ABTS^•+^) were determined according to the procedures reported by Alexandre et al. (38). The radical scavenging activity of DPPH was determined by measuring the absorbance at 517 nm (OD_517_). The scavenging activity of ABTS was determined by measuring the absorbance at 734 nm (OD_734_). The radical scavenging activity of superoxide was determined according to the procedures reported by Jing and Zhao (39). The absorbance was measured at 420 nm (OD_420_). The scavenging activities of hydroxyl radical (^•^OH) and superoxide radical (O_2_^•−^) were determined using commercial kits according to the manufacturer’s instructions (Nanjing Jiancheng Institute of Bioengineering, Nanjing, China). The results are expressed as units (U) per milligram of protein for ^•^OH ^−^ and U per gram of protein for O_2_^•−^.

### 2.8 Histological analysis

Fixed tissues were dehydrated, cleared, and embedded in paraffin. Paraffin sections (5 μm) were stained with hematoxylin and eosin. All samples were viewed under a light microscope. Images were captured using an Olympus DP72 camera and DP controller software (version 2.2, DP2-BSW; Olympus Optical Co.) at 100× magnification, and two images of each slice were taken at 100 × field of view. Villus height (μm), crypt depth (μm), and villus height to crypt depth (V/C) ratio were calculated for 10 villi per bird and averaged to yield a single value for each broiler.

### 2.9 Visceral indices

In Experiment 2, bird body weight was recorded on day 42 after 12 h of feed but not water deprivation. One bird from each replication was euthanized by cervical dislocation (six birds per treatment). The heart, liver, spleen, lungs, and kidneys were wet-weighed immediately after dissection. All the organs were checked to ensure that they were not contaminated with blood clots or other materials. Visceral indices were calculated as the weight of visceral tissue (mg) divided by body weight (kg).

### 2.10 Statistical analysis

Normality and homogeneity of variance were checked using the normal probability plot and the residual plot of the JMP software program (JMP^®^, version 14; SAS Institute Inc., Cary, NC, United States). Differences between treatments were determined using Analysis of Variance (ANOVA) in JMP 14.0. Mean separations were achieved using the Tukey HSD test at 5%. Errors are expressed as Standard Errors of the Mean (SEM). Regression analysis was used to test the linear and quadratic effects of increasing levels of PFAC supplementation. Differences were regarded as statistically significant at *p* < 0.05; an indicative trend was defined as 0.05 ≤ *p* < 0.10.

A multivariate correlation analysis was performed on all the data collected from all treatments. Intestinal MDA, IL-1β, IFN-γ, SIgA, and IGF-I levels were variables used to calculate Pearson’s correlation coefficients. Scatterplot matrices of the correlations were graphed. For discussion of the relative strength of the correlation, *r* was defined as follows (40): very weak (∣*r*∣ < 0.200), weak (∣*r*∣ = 0.200–0.399), moderate (∣*r*∣ = 0.400–0.599), strong (∣*r*∣ = 0.600–0.799), and very strong (∣*r*∣ = 0.800–0.999). Differences were regarded as statistically significant at *p* < 0.01; an indicative trend was defined as 0.01 ≤ *p* < 0.05; a potential correlation was defined as *p* < 0.10.

## 3 Results

### 3.1 Growth performance

From d 1 to d 21, dietary supplementation with 750 mg/kg and 1,000 mg/kg of PFAC increased (*p* < 0.05) both BWG and FI compared to CON (Table 2), while activated charcoal at 450 mg/kg did not alter BWG or FI in either phase. G:F was not significantly affected by either PFAC or activated charcoal during the experiment (*p* > 0.05).

**Table 2:** Effect of dietary treatments on broiler growth performance.^a^

Item^a^	AC	CON	PFAC, mg/kg				SEM	*p*-value	Linear	Quadratic
			250	500	750	1,000				
Body weight, g										
1 d	41.51	42.00	41.69	41.86	41.68	41.69	0.1	0.71	0.81	0.84
21 d	551.53^c^	549.51^c^	552.00^c^	564.14^bc^	614.60^ab^	610.70^ab^	4.3	0.04	0.12	0.08
42 d	1855.70	1861.14	1880.22	1906.30	1932.88	1916.21	22.16	0.09	0.03	0.13
1–21 d										
BWG, g/bird	510.88^c^	508.72^c^	512.31^c^	523.82^bc^	578.24^c^	571.89^ab^	5.8	0.04	0.72	0.09
FI, g/bird	790.22^b^	782.41^b^	771.81^b^	788.53^b^	834.90^a^	836.61^a^	10.1	<0.01	0.70	0.29
G: F	0.64	0.65	0.67	0.67	0.69	0.68	0.02	0.54	0.69	0.36
22–42 d										
BWG, g/bird	1290.13	1283.41	1310.64	1342.70	1329.40	1309.69	18.1	0.06	0.01	0.09
FI, g/bird	2342.8	2248.7	2257.4	2272.9	2201.88	2198.31	29.4	0.25	0.09	0.20
G: F	0.55	0.57	0.58	0.59	0.61	0.60	0.12	0.25	0.14	0.37
1–42 d										
BWG, g/bird	1801.12	1792.11	1822.90	1866.52	1907.61	1881.59	0.47	0.05	0.02	0.04
FI, g/bird	3133.67	3031.14	3029.21	3061.44	3036.83	3034.90	0.50	0.49	0.40	0.15
G: F	0.57	0.59	0.60	0.61	0.63	0.62	0.10	0.38	0.14	0.54

a Dietary treatments were PFAC supplementation at doses of 0, 250, 500, 750, and 1,000 mg/kg (CON, PFAC250, PFAC500, PFAC750, and PFAC1000) and 450 mg/kg activated charcoal (AC) supplementation. Differences among all dietary treatments were analyzed by ANOVA. Significant differences between groups were expressed as different superscript capital (*p* < 0.05) or lowercase (*p* < 0.01) letters in the same row. Linear and quadratic analyses were performed between CON and PFAC treatments. SEM, standard error of the mean; BWG, body-weight gain; FI, feed intake; G:F, gain-to-feed ratio.

### 3.2 Protein, lipid, and glucose metabolism

**Figure 1:**
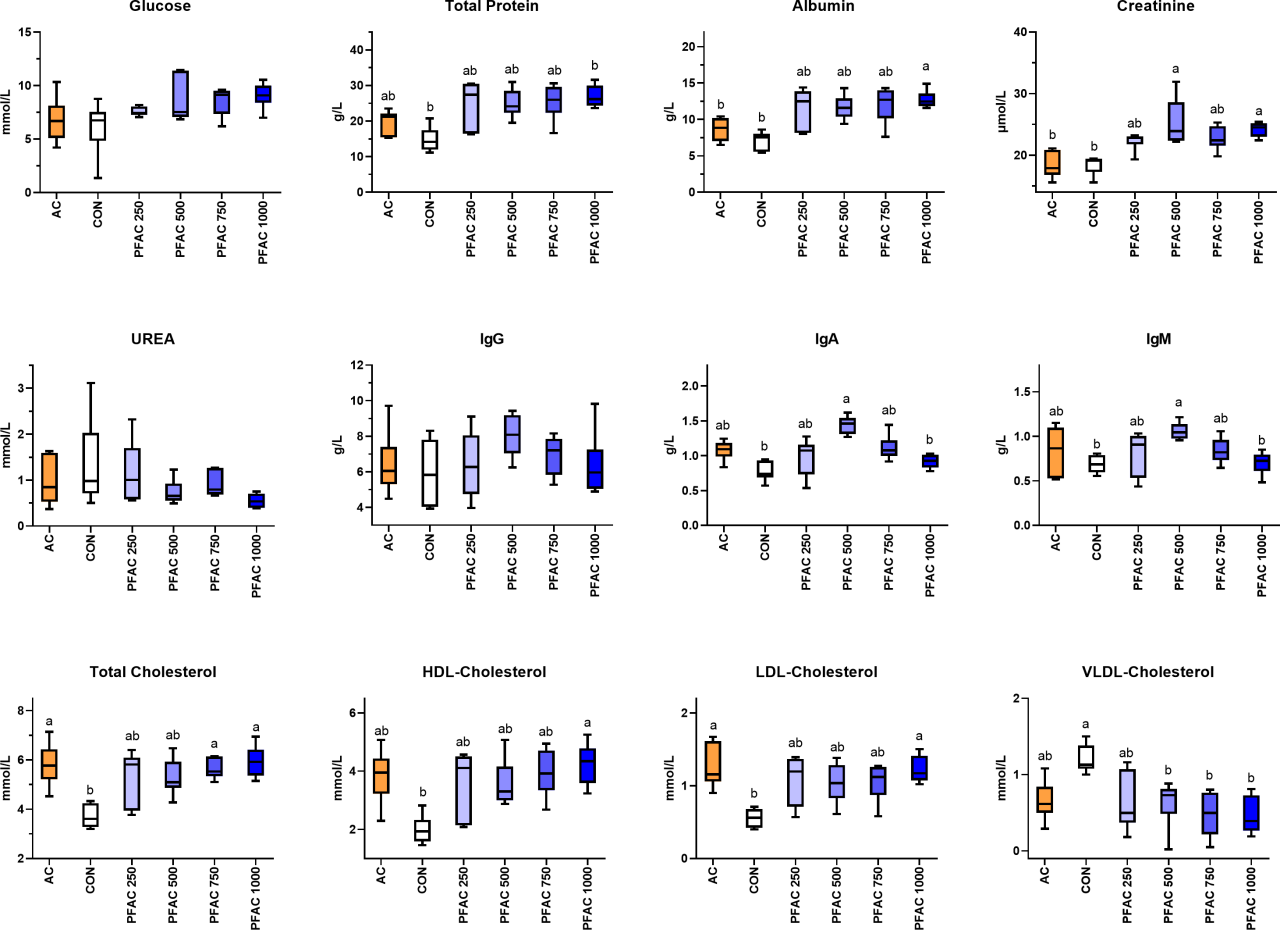
Effects of PFAC on serum biochemical indices in broilers on d 21. Dietary treatments were PFAC supplementation at the doses of 0, 250, 500, 750, and 1,000 mg/kg diets (CON, PFAC250, PFAC500, PFAC750, and PFAC1000) and 450 mg/kg activated charcoal (AC) supplementation. Boxes with different letters indicate significant differences between treatments at the level of *p* < 0.05.

PFAC treatments increased total protein (TP), and PFAC treatments at 1000 mg/kg increased albumin content compared to CON and AC at d 21 (*p* < 0.05) (Figure 1). Similarly, serum creatinine levels were increased by PFAC supplementation at 500 mg/kg and 1,000 mg/kg at d 21 compared to CON and AC (*p* < 0.01). These alterations in glucose and protein metabolism by PFAC supplementation were not observed on d 42, except that PFAC supplemented at 1000 mg/kg decreased serum urea concentration (*p* < 0.05). PFAC treatments generally had no significant effect on serum glucose on d 21 and d 42. PFAC treatments from 250 to 1,000 mg/kg increased serum HDL-cholesterol and LDL-cholesterol levels but decreased VLDL-cholesterol levels on d 21 compared to CON (*p* < 0.01) (Figure 1). This effect was comparable to the activated charcoal treatment. On d 42, all treatments, including PFAC at all dosages and activated charcoal, had no significant effect on serum cholesterol in broilers (*p* > 0.05).

### 3.3 Intestinal morphology

PFAC treatment generally improved intestinal morphology (Table 3). In the ileum, PFAC at 500 mg/kg increased villus height compared to CON (*p* < 0.05). Compared to CON, PFAC at 750 mg/kg increased villus height in the duodenum and jejunum (*p* < 0.05). Villus height in the duodenum showed a quadratic response to PFAC doses (*p* < 0.05). Crypt depth and the ratio of villus height to crypt depth were not significantly altered by PFAC. Villus height, crypt depth, and their ratio in the intestine showed no significant response to activated charcoal treatment.

**Table 3:** Effect of dietary treatments on broiler intestinal morphology.^a^

Item^a^	AC	CON	PFAC, mg/kg				SEM	*P*-value	Linear	Quadratic
			250	500	750	1,000				
Duodenum										
Villus height, μm	1250.60^b^	1270.04^b^	1296.43^b^	1520.24^ab^	1701.21^a^	1567.52^ab^	12.10	<0.01	0.61	0.04
Crypt depth, μm	164.37	161.36	189.49	182.62	194.00	184.63	9.82	0.34	0.87	0.08
V/C	8.20	8.67	6.58	9.07	8.59	8.52	1.22	0.09	0.89	0.48
Jejunum										
Villus height, μm	1209.60^c^	1200.61^c^	1293.19^b^	1440.48^a^	1427.75^a^	1334.66^b^	34.63	0.04	0.06	0.09
Crypt depth, μm	148.30	133.54	139.63	152.99	150.14	145.79	3.47	0.11	0.07	0.15
V/C	8.16^b^	8.36^b^	8.74^b^	8.54^b^	8.83^b^	9.05^a^	20.38	0.04	0.11	0.94
Ileum										
Villus height, μm	1204.57^b^	1221.06^b^	1285.07^b^	1329.50^a^	1161.58^b^	1176.35^b^	19.86	0.03	0.10	0.12
Crypt depth, μm	179.36	174.72	182.83	192.67	170.28	185.43	7.18	0.06	0.19	0.27
V/C	6.72	6.84	7.18	6.86	6.47	6.23	1.03	0.15	0.57	0.57

a Dietary treatments were PFAC supplementation at doses of 0, 250, 500, 750, and 1,000 mg/kg (CON, PFAC250, PFAC500, PFAC750, and PFAC1000) and 450 mg/kg activated charcoal (AC) supplementation. Differences among all dietary treatments were analyzed by ANOVA. Significant differences between groups were expressed as different superscript capital (*p* < 0.05) or lowercase (*p* < 0.01) letters in the same row. Linear and quadratic analyses were performed between CON and PFAC treatments. SEM, standard error of the mean; V/C, the ratio of villus height to crypt depth.

### 3.4 Immunoglobulins and antioxidant indices

**Figure 2:**
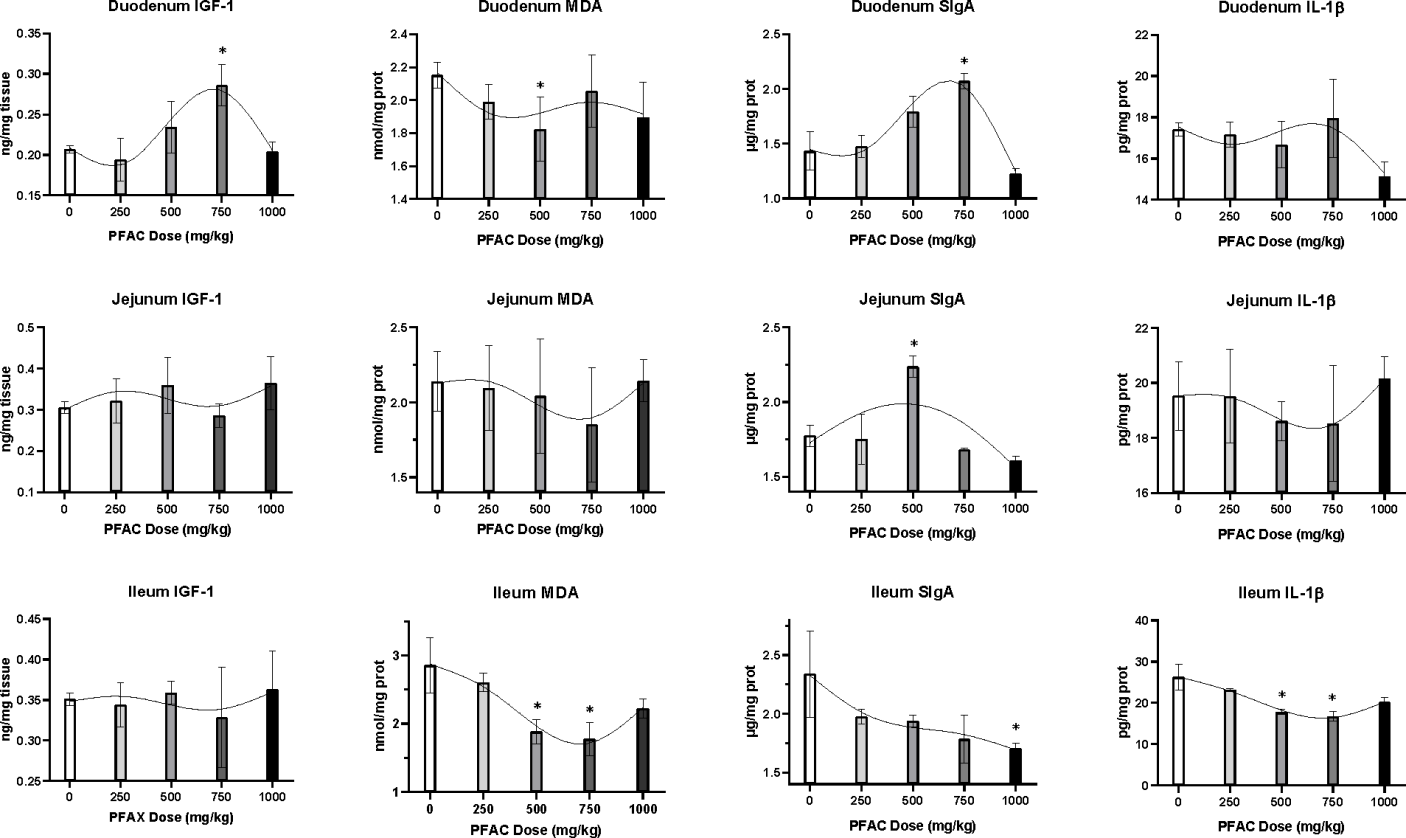
Effects of PFAC on indices related to local growth (IFG-1), antioxidant (MDA), immunity (SIgA), and inflammation (IL-1β) of intestine in broilers on d 42. Dietary treatments were PFAC supplementation at doses of 0, 250, 500, 750, and 1,000 mg/kg. Dietary activated charcoal (AC) at 450 mg/kg was also included in the experiment but is not shown in the figure. The results of the AC treatment showed no difference from the unsupplemented treatment (*p* > 0.05). The results of different doses of PFAC were analyzed by ANOVA, and * indicates a significant difference from the unsupplemented treatment at the level of *p* < 0.05. GraphPad Prism (8.0) was used to analyze the data, and a restricted cubic spline curve was generated to visualize the relationship between predicted intestinal indices and PFAC doses in broilers.

On d 21, PFAC treatments at 500 mg/kg increased serum IgA and IgM levels compared to CON (*p* < 0.05) (Figure 1). Activated charcoal did not alter serum immunoglobulins compared to CON. As shown in Figure 2, PFAC at 500 mg/kg decreased MDA levels in the duodenum and ileum compared to CON (*p* < 0.05). PFAC treatments at 750 mg/kg increased SIgA levels in the duodenum and decreased IL-1β levels in the ileum (*p* < 0.05).

### 3.5 Effect of PFAC on scavenging activities in muscle

Significant differences were found in the scavenging activities of DPPH, ABTS^•+^, and O_2_^•−^ attributable to the dietary treatments compared to CON and AC (Table 4). The DPPH-scavenging activity of breast muscle in PFAC-treated broilers was greater than that of CON (*p* < 0.05). Dietary supplementation of 500 mg/kg PFAC had higher ABST^•+^ reducing activities in the breast and thigh than CON (*p* < 0.05). In the thigh, dietary PFAC in broiler feed demonstrated an increased ability to scavenge O_2_^•−^ free radicals compared with CON (*p* < 0.05).

**Table 4:** Effect of dietary treatments on the free radical scavenging activity of broiler meat.^a^

Item^a^	AC	CON	PFAC, mg/kg				SEM	*p*-value
			250	500	750	1,000		
Breast								
DPPH, %	24.22^a^	23.49^a^	29.98^c^	30.44^c^	27.91^b^	27.31^b^	0.57	<0.01
ABTS^•+^, %	33.49^a^	30.67^a^	41.75^ab^	45.05^b^	44.22^b^	40.66^ab^	1.07	<0.01
O^•^, U/g protein	1.01^a^	0.98^a^	1.08^b^	1.18^c^	1.04^b^	1.03^b^	0.02	<0.01
OH^•^, U/mg protein	17.61	16.92	18.22	18.23	18.01	18.29	0.23	0.51
Thigh								
DPPH, %	21.13	21.11	23.26	21.08	21.11	22.10	0.32	0.53
ABTS^•+^, %	30.58^a^	29.94^a^	34.12^b^	32.46^b^	29.94^a^	32.04^b^	0.45	0.02
O^•^, U/g protein	1.28^a^	1.27^a^	1.33^ab^	1.41^b^	1.21^a^	1.29^a^	0.02	0.01
OH^•^, U/mg protein	20.42	19.70	20.09	21.40	19.93	20.15	0.28	0.62

a Dietary treatments were PFAC supplementation at doses of 0, 250, 500, 750, and 1,000 mg/kg (CON, PFAC250, PFAC500, PFAC750, and PFAC1000) and 450 mg/kg activated charcoal (AC) supplementation. Differences among all dietary treatments were analyzed by ANOVA. Significant differences between groups were expressed as different superscript capital (*p* < 0.05) or lowercase (*p* < 0.01) letters in the same row. Linear and quadratic analyses were performed between CON and PFAC treatments. SEM, standard error of the mean; ABTS^•+^, (2,2′-azino-bis (3-ethylbenzthiazoline-6-sulfonic acid)), DPPH, 2,2-diphenyl-1-picrylhydrazyl.

### 3.6 Correlation analysis of antioxidant and inflammatory parameters

**Figure 3:**
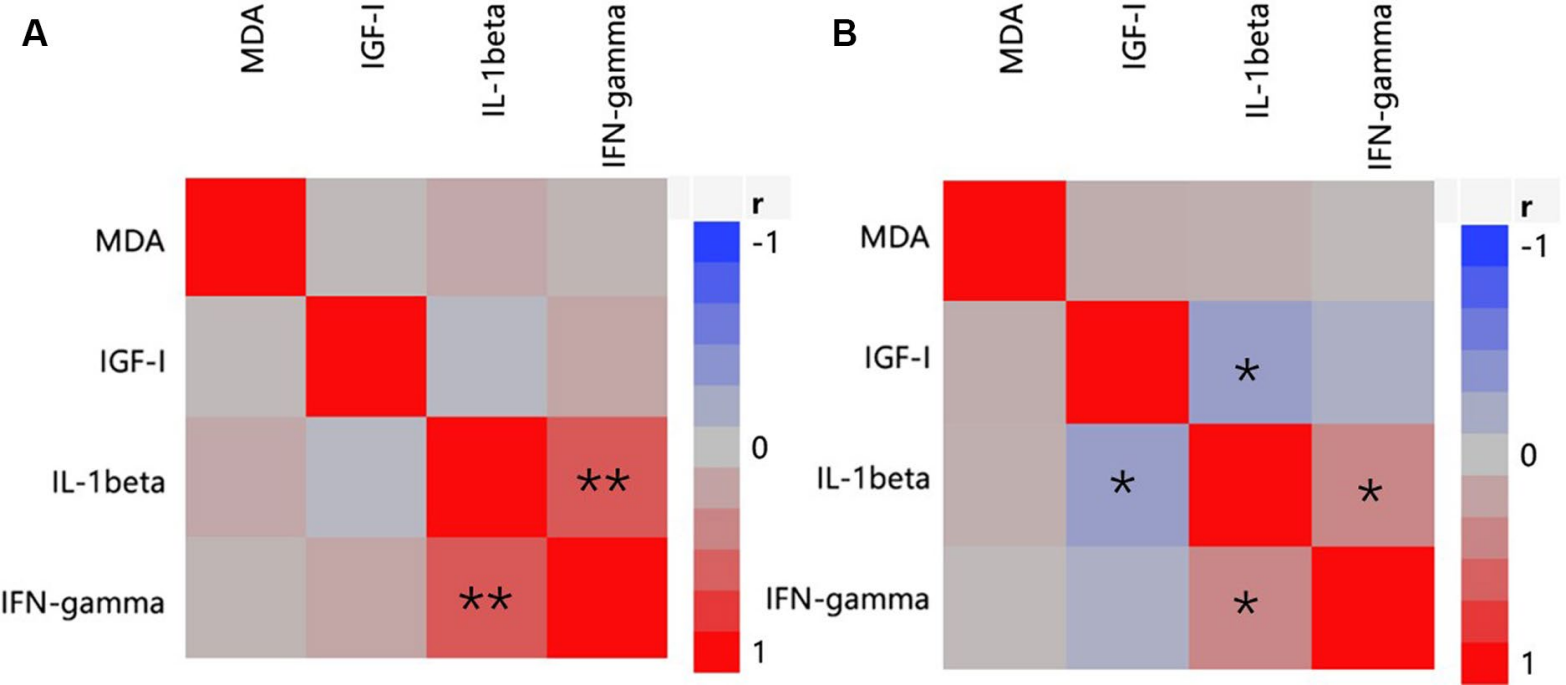
Heat map of correlation analysis on serum indices of oxidative stress, antioxidant, inflammatory, and growth factors represented by concentrations of MDA, concentrations of IL-1β and IFN-γ, and IGF-I. **(A)** At d 21; **(B)** at d 42. Color indicates correlation coefficient, *r*; blue indicates negative correlation, and red indicates positive correlation. * indicates *p* < 0.05 and **indicates *p* < 0.01.

In the serum (Figure 3), to identify the relationship between antioxidants (represented by MDA), inflammatory parameters (represented by IL-1β, IFN-γ), and growth factors (represented by IGF-I), data from all treatments (CON, AC, and PFAC250, PFAC500, PFAC750, and PFAC1000) were collected, and multivariate correlation analysis was performed on these data. IL-1β levels were strongly and positively correlated with IFN-γ levels on d 21 (*r* = 0.642) and weakly correlated with IFN-γ levels on d 42 (*r* = 0.394) (*p* < 0.05). Serum IGF-I had no significant correlations with serum levels of MDA, IL-1β, and IFN-γ on d 21, but a weak negative correlation with IL-1β (*r* = −0.317) on d 42.

**Figure 4:**
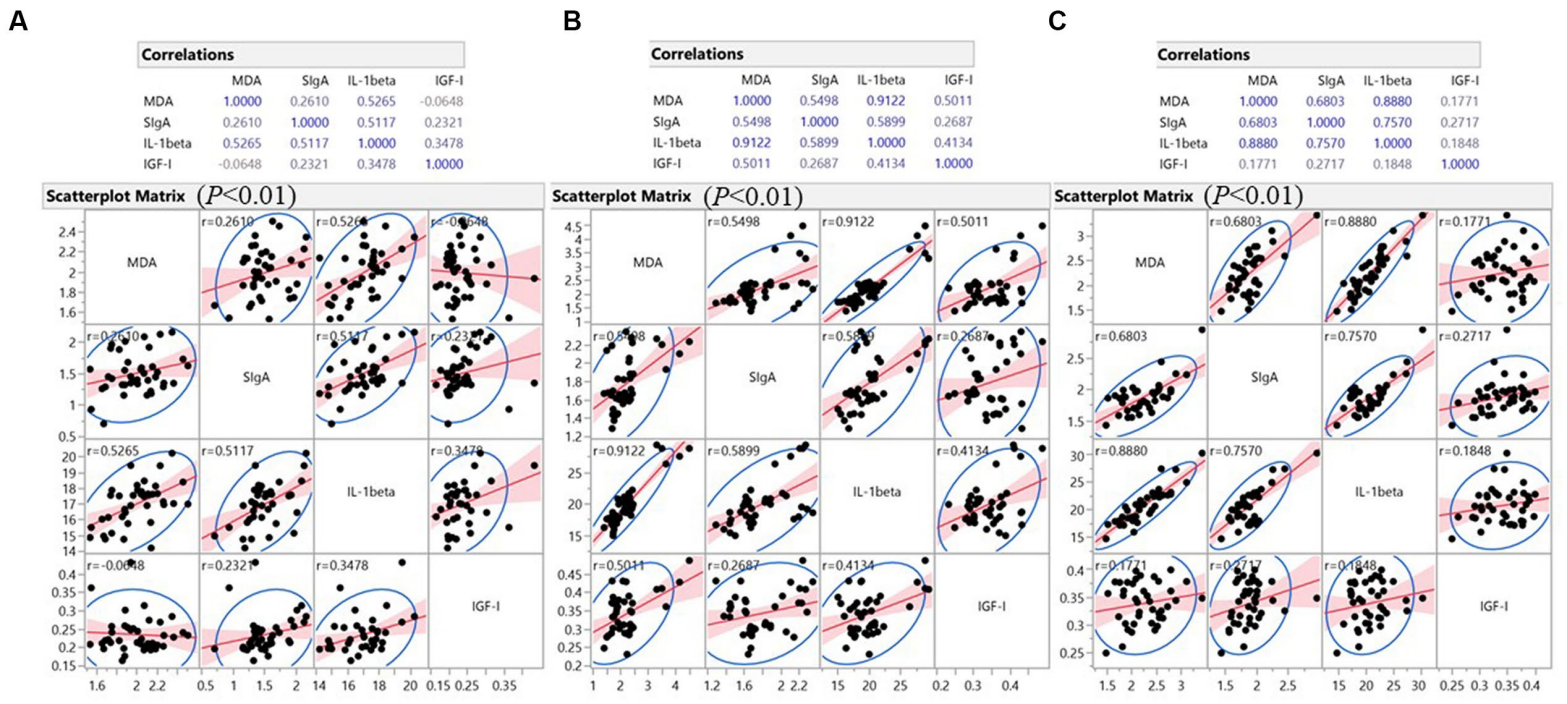
Pearson’s Correlation analysis was performed on all intestine data collected from six treatments. **(A)** Data analysis of duodenum; **(B)** Data analysis of jejunum; **(C)** Data analysis of ileum. The relative strength of the correlation was defined as follows: very weak (∣*r*∣ < 0.200), weak (∣*r*∣ = 0.200–0.399), moderate (∣*r*∣ = 0.400–0.599), strong (∣*r*∣ = 0.600–0.799), and very strong (∣*r*∣ = 0.800–0.999). Differences were considered statistically significant at *p* < 0.01; an indicative trend was defined as 0.01 ≤ *p* < 0.05; a potential correlation was defined as *p* < 0.10.

In the intestine (Figure 4), MDA, representing oxidative levels, showed moderate to very strong positive correlation (*p* < 0.01) with IL-1β levels (*r* = 0.527, 0.912, and 0.888, respectively) in the duodenum, jejunum, and ileum. In the jejunum and ileum, MDA levels also showed moderate to strong positive correlations (*p* < 0.01) with mucosal SIgA (*r* = 0.550 and 0.680, respectively). In all three intestinal segments, IL-1β and SIgA showed moderate to strong positive correlations (in duodenum, *r* = 0.512; in jejunum, *r* = 0.590; in ileum, *r* = 0.757). These observations suggest concurrent oxidative stress, an inflammatory response, and adaptive immunity in the intestine. Interestingly, in the jejunum, IGF-I had a moderate positive correlation with MDA (*r* = 0.501), IL-1β (*r* = 0.413), and a weak correlation with SIgA (*r* = 0.269) (*p* < 0.01). In the other two intestinal segments, this correlation is less significant, as IGF-I in the duodenum showed a trend of positive correlation with IL-1β (0.01 ≤ *p* < 0.05) and a potential positive correlation with SIgA in the ileum (*p* < 0.10). The results indicated a jejunum-specific positive correlation of local growth factors with inflammatory and immune factors.

### 3.7 Safety of PFAC in broilers

In the starter phase (Table 5), the addition of PFAC had no significant effect on the organ index of broilers compared with CON (*p* > 0.05). No visible histopathologic differences in the liver, spleen, or kidney were observed among the three treatments (data not shown).

**Table 5:** Effect of excess PFAC on broiler visceral index.^a^

Visceral Index, mg/kg	PFAC, mg/kg			SEM	*p*-value
	0	750	7,500		
d 21					
Heart	7.77	8.08	8.09	0.08	0.13
Liver	22.29	22.81	23.73	0.32	0.17
Spleen	0.94	0.81	0.83	0.03	0.12
Lung	5.78	5.08	5.40	0.14	0.13
Kidney	7.09	6.89	7.24	0.08	0.26
d 42					
Heart	5.33	5.39	5.53	0.09	0.72
Liver	15.94	16.15	15.97	0.24	0.95
Spleen	0.86	0.82	1.01	0.04	0.06
Lung	4.32	4.29	4.28	0.03	0.83
Kidney	6.24	5.94	5.84	0.10	0.24

a Dietary treatments were PFAC supplementation at doses of 0, 750, and 7,500 mg/kg. Differences among all dietary treatments were analyzed by ANOVA. SEM, standard error of the mean.

On d 21, dietary supplementation with 7,500 mg/kg PFAC decreased the number of lymphocytes compared with the 750 mg/kg PFAC treatment (*p* < 0.05), and the platelet distribution width (PDW) of 750 mg/kg PFAC did not differ from that of 7,500 mg/kg PFAC (Table 6). On d 42, supplementation with 7,500 mg/kg PFAC had higher numbers of lymphocytes and lower PDW compared with that of 750 mg/kg PFAC (*p* < 0.05).

**Table 6:** Effect of excess PFAC on blood biochemical parameters in broilers.^a^

Item^a^	PFAC, mg/kg			SEM	*p*-value
	0	750	7,500		
d 21					
Erythrocyte indices					
RBC, 10^12^/L	2.54	2.56	2.60	0.05	0.46
HGB, g/L	104.26	104.80	108.41	2.06	0.39
HCT, L/L	0.25	0.27	0.28	0.01	0.06
MCV, fL	106.99^b^	108.97^a^	109.44^a^	0.69	0.01
MCH, pg	40.13	41.04	42.08	0.28	0.53
MCHC, g/L	347.50	360.10	358.67	1.91	0.82
Leukocyte indices, 10^9^/L					
Leukocytes	155.39	157.95	153.48	0.72	0.07
Lymphocytes	66.44^a^	65.52^a^	57.77^b^	1.15	0.03
Granulocytes	63.32	67.19	65.88	1.42	0.12
Platelet indices					
PLT, 10^9^/L	34.07	30.03	29.91	1.43	0.09
PCT, L/L	0.05	0.04	0.04	0.01	0.10
PDW, %	41.95^b^	50.11^a^	53.05^a^	2.26	<0.05
MPV, fL	15.53	14.62	15.00	0.05	0.86
d 42					
Erythrocyte indices					
RBC, 10^12^/L	2.97^a^	2.60^ab^	2.52^b^	0.04	<0.01
HGB, g/L	110.75	112.80	108.76	1.44	0.34
HCT, L/L	0.31^a^	0.27^ab^	0.24^b^	0.01	0.02
MCV, fL	107.02	110.18	107.93	0.48	0.59
MCH, pg	37.72	39.40	42.08	0.40	0.22
MCHC, g/L	377.00	381.00	388.00	3.17	0.32
Leukocyte indices 10^9^/L					
Leukocytes	159.60	157.28	161.55	0.77	0.39
Lymphocytes	50.48^b^	51.16^b^	55.24^a^	1.23	<0.01
Granulocytes	86.71^a^	84.54^ab^	80.75^b^	1.70	0.03
Platelet indices					
PLT, 10^9^/L	38.06^b^	46.42^a^	47.00^a^	2.03	0.01
PCT, L/L	0.04^b^	0.05^a^	0.06^a^	0.01	0.01
PDW, %	50.04^a^	44.60^b^	41.34^c^	2.26	<0.01
MPV, fL	12.09	12.78	12.99	0.06	0.14

a Dietary treatments were PFAC supplementation at doses of 0, 750, and 7,500 mg/kg. Differences among all dietary treatments were analyzed by ANOVA.

b The significant differences between groups were expressed as different superscript capital (*p* < 0.05) or lowercase (*p* < 0.01) letters in the same row. SEM, standard error of the mean. Red blood cell count, RBC; hemoglobin, HGB; hematocrit, HCT; mean corpuscular volume, MCV; mean corpuscular hemoglobin, MCH; mean corpuscular hemoglobin concentration, MCHC; platelet count, PLT; plateletcrit, PCT; Platelet distribution width, PDW; mean platelet volume, MPV.

**Figure 5:**
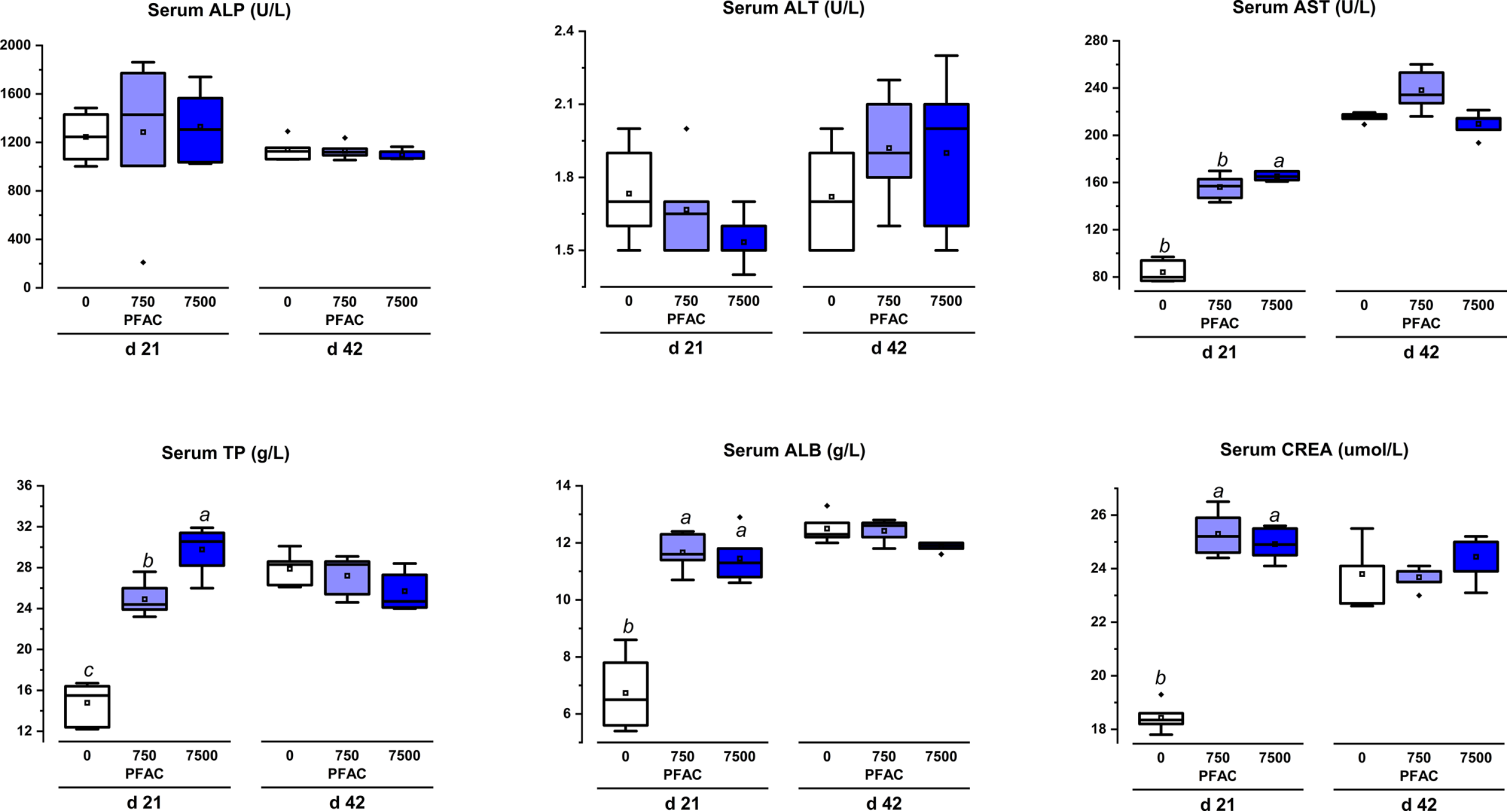
Effect of excess levels of PFAC on serum parameters in broilers on d 21 and d 42. Dietary treatments were PFAC supplementation at doses of 0, 750, and 7,500 mg/kg. ALP, alkaline phosphatase; ALT, alanine aminotransferase; AST, aspartate aminotransferase; TP, total protein, ALB, albumin; CREA, creatinine. Bars with different letters indicate significant differences (*p* < 0.05). Differences among all dietary treatments were analyzed by ANOVA. Means in the same row with different letters indicate statistical differences at the level of *p* < 0.05. SEM, standard error of the mean.

On d 21 and d 42, the dietary supplementation of PFAC (7,500 mg/kg) did not show differences from the treatment of the 750 mg/kg PFAC supplementation group on the indices of AST, ALT, and ALP, which represent liver damage (Figure 5). Dietary supplementation of 7,500 mg/kg PFAC significantly increased serum TP and ALB on d 21 compared to the unsupplemented treatment (*p* < 0.05) but showed no differences with the treatment of 750 mg/kg PFAC. Dietary supplementation of PFAC had no significant effect on the serum biochemistry of broiler chickens on d 42 (*p* > 0.05).

## 4 Discussion

Chinese herbal extracts contain various active components that exert antibacterial, anti-inflammatory, and antioxidant activities. These active components include phenolic acids, flavonoids, alkaloids, saponins, and polysaccharides. Extraction of herbs with alcohol contains more total phenolic acids and total flavonoids than aqueous extraction (41). In our previous studies, herbal extract was coupled with activated charcoal to form a charcoal-herb complex for feeding piglets and broilers. We found that the charcoal-herb complex promoted growth performance, enhanced body immunity, reduced inflammation, and improved antioxidant status (29–32). Four types of Chinese medicine were used according to their specific functions reported in the scientific literature (42–45). These extracts from mixed medicinal herbs were not effective when fed at 25 mg/kg in feed (32). This dose was more than 200 times lower than previously reported beneficial doses of mixed extracts (46). However, this low dose of herbal extract significantly facilitated the function of activated charcoal in promoting growth performance, anti-inflammatory, and antioxidant activities (32). Without sorption of the extract, activated charcoal at the same dose showed no such activity. In our studies, the herbs were extracted with either aqueous (32) or alcoholic (30) methods, and they showed similar positive effects on the facilitation of activated charcoal in broilers. These results raised the question of whether the herbal extract, regardless of the extraction method, contained major components that could enhance the activity of activated charcoal.

In the present study, we found that AC sorption of the herb extract containing more than 80% of phenols and flavonoids was beneficial to the growth performance of broilers (at 750 or 1,000 mg/kg PFAC) and the improvement of anti-inflammatory and antioxidant status (at 500 or 750 mg/kg PFAC) in the ileum of broilers. These results were similar to those of activated charcoal sorption of the herbal extract of four types of medicinal Chinese herbs (28). These indicated that phenols and flavonoids represented the majority of the functions of herbal extracts in cooperating with activated charcoal. In the literature, wood vinegar containing organic acids and phenols, with activated charcoal, effectively improved growth performance, intestinal morphology, and antimicrobial activity in broilers (47–50).

Coating charcoal with liquid organic substances can alter the chemical properties of charcoal by improving its hydrophilicity, which is beneficial in the aqueous environment of the digestive tract (25). In addition to adsorption activity, charcoal or activated charcoal can act as geo-batteries because it can accept, store, and mediate electrons in biochemical reactions. Surface ligands such as phenol can harbor and aid electron transfer (23). Herbal extracts usually contain multiple active components, such as phenols and flavonoids (51). Phenols are active ligands of the retinoid X receptor, and flavonoids are ligands to activate the pregnane X receptor. Retinoid X receptors can form heterodimers with multiple nuclear receptors to initiate a variety of physiological and pharmacological signaling pathways (52). The Pregnane X receptor is a master transcription factor for xenobiotic-induced expression of key genes encoding metabolic enzymes and transporters (53). Phenols and flavonoids represent a majority of the functions of the herbal extract, regardless of the specific herbs sourced, in cooperation with activated charcoal in promoting growth performance and improving anti-inflammatory and antioxidative status in broilers, as proved in this study. The results of this study were very useful for the production of activated charcoal and the sorption of herbal extracts. The contents of phenols and flavonoids were more important than the number and types of medicinal herbal extracts. In one of our previous studies, four types of Chinese herbs were used for herbal extract, including *Pulsatilla chinensis*, *Portulaca oleracea L.*, *Artemisia argyi Folium*, and *Pteris multifida Poir*. Among them, *Portulaca oleracea L*. is more abundant and low-cost. In this study, the phenols and flavonoids from the herbal extract of *Portulaca oleracea L*., sorption of activated charcoal received similar promoting effects compared with those of the extracts from the four types of herbs. The results of this study could significantly reduce the cost of producing activated charcoal sorption herbal extract.

The present study on broilers confirmed the finding that supplementing feed with PFAC could induce a positive effect on cholesterol production compared to CON (31), but not with AC. Although the serum biochemistry differed among the treatments, the values were all within the normal reference range for broilers (54, 55). Chinese herbal extract contains polyphenol, which induces a reduction in total cholesterol and LDL-C by increasing bile acid excretion (56). The inclusion of PFAC had a greater effect on broilers in the starter phase, with increased lipid metabolism, than in the grower phase. Other researchers have suggested that the response of broilers to dietary charcoal may vary with age (57, 58).

The production, utilization, and scavenging of reactive oxygen species (ROS) in the body maintain a dynamic balance. When the body’s ROS production and antioxidant system are out of balance, oxidative stress occurs, leading to the overproduction of lipids, proteins, nucleic acids, and other macromolecules in the body (59). The levels and activities of antioxidant enzymes such as total SOD, GSH-PX, and CAT are often used as important indicators to evaluate the function of body tissues and organs (60). The results of this experiment demonstrated that dietary supplementation with PFAC could increase the antioxidant capacity of broilers by improving total SOD activity in the liver and kidney, as reported by Jiang et al. (61). The oxidative stability of chickens is related to the free radical scavenging capacity of muscle tissue and dietary antioxidant content (62).

Oxidative stress and inflammatory responses are often associated with enteritis in broilers and piglets (3, 4). If an antioxidant can alleviate structural damage to the intestine induced by oxidative stress and the inflammation-induced immune response, it is logical to propose that antioxidants can save energy for animals to redirect to improved growth performance. Therefore, we conducted an analysis of all specimens of the study treatments on the correlations between oxidative stress, antioxidant capacity, immune response, inflammatory response, and local growth factor. In the serum, we observed strong correlations in the broilers between the levels of MDA and IGF-1, and IL-1β and IFN-γ on d 21. There are few reports of a direct relationship between IL-1β and IFN-γ in the scientific literature. One of the links between them was contributed by the cells that secreted them, the B cells. IL-1β is a growth factor for B cell proliferation (63), while B cells can produce IFN-γ (64). In the intestine, IL-1β showed moderate to very strong positive correlations with MDA levels and SIgA levels. This was evidence of concurrent lipid peroxidation and the occurrence of local inflammation in the intestine. Oxidative stress induced by toxins or pathogens can cause inflammation and mucosal immune responses (65, 66). For example, oxidized low-density lipoprotein induced adhesion, influx of monocytes, and cytokine release (67). Peyer’s patches, which harbor B cells, T cells, macrophages, and dendritic cells located in the jejunum and ileum, greatly impact both cellular and humoral immunity in the intestine (68). Interestingly, serum IGF-I level was negatively correlated with serum IL-1β, while IGF-I level had a weak positive correlation with IL-1β and SIgA in the jejunum. This result revealed different roles of IGF-I in the systemic circulation. IGF-I may be related to body growth and local release from intestinal macrophages, which may be involved in tissue repair. Macrophages involved in wound repair increased IGF-I production to help alleviate local tissue damage caused by oxidative stress (69, 70). Correlation analysis revealed a scenario of antioxidant, anti-inflammatory, and promoted growth effects in the jejunum and ileum.

In clinical practice, serum albumin levels are associated with granular degeneration of the liver and are considered to be an important indicator of liver disease (71, 72). The addition of PFAC caused an increase in these serum biochemical parameters, but the values obtained in this test were within the range of normal poultry blood biochemistry (54, 73). Furthermore, no pathological changes were observed in histological sections of the liver and kidney. These results suggest that PFAC supplementation up to 7,500 mg/kg in broiler diets has no detrimental effects on organ and body health, which may be related to the increased antioxidant capacity of the organism.

## 5 Conclusion

In conclusion, the sorption of phenols and flavonoids into activated charcoal (500 mg/kg to 1,000 mg/kg) improved growth performance, anti-inflammatory and antioxidant status, protein metabolism, and intestinal morphology. Concerning these indices, phenols, and flavonoids, in cooperation with activated charcoal, represented a majority of the functions of the herbal extracts from multiple Chinese medicinal herbs. Excessive PFAC (7,500 mg/kg) showed no significant detrimental effects on broiler growth, liver function, or hematology. These results suggest that PFAC is a safe feed additive for broilers.

## Data availability statement

The original contributions presented in the study are included in the article/supplementary material, further inquiries can be directed to the corresponding author.

## Ethics statement

The animal studies were approved by China Agricultural University Institutional Animal Care and Use Committee. The studies were conducted in accordance with the local legislation and institutional requirements. Written informed consent was obtained from the owners for the participation of their animals in this study.

## Author contributions

YZ: Conceptualization, Data curation, Writing – original draft. XF: Methodology, Writing – review & editing. LW: Conceptualization, Data curation, Formal analysis, Writing – review & editing. XG: Conceptualization, Data curation, Investigation, Writing – review & editing. BD: Investigation, Methodology, Writing – review & editing.

## References

[1] Hughes R. An integrated approach to understanding gut function and gut health of chickens. Asia Pac J Clin Nutr. (2005) 14:S27.

[2] Ochieng PE Scippo M-L Kemboi DC Croubels S Okoth S. Mycotoxins in poultry feed and feed ingredients from sub-Saharan Africa and their impact on the production of broiler and layer chickens: a review. Toxins. (2021) 13:633. doi: 10.3390/toxins13090633, PMID: 34564637 PMC8473361

[3] Akbarian A Michiels J Degroote J Majdeddin M Golian A. Association between heat stress and oxidative stress in poultry; mitochondrial dysfunction and dietary interventions with phytochemicals. J Anim Sci Biotechnol. (2016) 7:37. doi: 10.1186/s40104-016-0097-5, PMID: 27354915 PMC4924307

[4] Adams CA. Nutrition-based health in animal production. Nutr Res Rev. (2006) 19:79–89. doi: 10.1079/NRR2005115, PMID: 19079877

[5] Anadón A. Ws14 the Eu ban of antibiotics as feed additives (2006): alternatives and consumer safety. J Vet Pharmacol Ther. (2006) 29:41–4. doi: 10.1111/j.1365-2885.2006.00775_2.x16420301

[6] Ministry of Agriculture and rural Affairs of People’s Republic of China, vol. 258 (2020).

[7] Zhang J Liu J Liu R. Effects of pyrolysis temperature and heating time on biochar obtained from the pyrolysis of straw and lignosulfonate. Bioresour Technol. (2015) 176:288–91. doi: 10.1016/j.biortech.2014.11.011, PMID: 25435066

[8] Zhou M. Application effect of charcoal in animal castration. Contemp Anim Husb. (2013) 8:59–60.

[9] Wu Y Ni S Yang B. Overview of Pharmic research on Chinese herbal-charcoal. Strait Pharm J. (2010):83–4.

[10] Holanda DM Kim SW. Mycotoxin occurrence, toxicity, and detoxifying agents in pig production with an emphasis on Deoxynivalenol. Toxins. (2021) 13:171. doi: 10.3390/toxins13020171, PMID: 33672250 PMC7927007

[11] Hatch RC Clark JD Jain AV Weiss R. Induced acute Aflatoxicosis in goats: treatment with activated charcoal or dual combinations of Oxytetracycline, Stanozolol, and activated charcoal. Am J Vet Res. (1982) 43:644–8. PMID: 6803625

[12] Burchacka E Lukaszewicz M Kulazynski M. Determination of mechanisms of action of active carbons as a feed additive. Bioorg Chem. (2019) 93:102804. doi: 10.1016/j.bioorg.2019.02.029 30782400

[13] Pirarat N Boonananthanasarn S Krongpong L Katagiri T Maita M. Effect of activated charcoal-supplemented diet on growth performance and intestinal morphology of Nile Tilapia (Oreochromis Niloticus). Thai J Vet Med. (2015) 45:113. doi: 10.56808/2985-1130.2615

[14] Ilomuanya M Ifudu N Uboh C. The use of metronidazole and activated charcoal in the treatment of diarrhea caused by Escherichia Coli 0157: H7 in an in vitro Pharmacodynamic model. Afr J Pharm Pharmacol. (2011) 5:1292–6. doi: 10.5897/AJPP11.274

[15] Senderovich H Vierhout MJ. Is there a role for charcoal in palliative diarrhea management? Curr Med Res Opin. (2018) 34:1253–9. doi: 10.1080/03007995.2017.1416345, PMID: 29231746

[16] Naka K Watarai S Inoue K Kodama Y Oguma K. Adsorption effect of activated charcoal on Enterohemorrhagic Escherichia Coli. J Vet Med Sci. (2001) 63:281–5. doi: 10.1292/jvms.63.281, PMID: 11307928

[17] Chu GM Kim JH Kim HY Ha JH Jung MS Song Y. Effects of bamboo charcoal on the growth performance, blood characteristics and noxious gas emission in fattening pigs. J Appl Anim Res. (2013) 41:48–55. doi: 10.1080/09712119.2012.738219

[18] Kana JR Teguia A Mungfu BM Tchoumboue J. Growth performance and carcass characteristics of broiler chickens fed diets supplemented with graded levels of charcoal from maize cob or seed of Canarium Schweinfurthii Engl. Trop Anim Health Pro. (2011) 43:51–6. doi: 10.1007/s11250-010-9653-8, PMID: 20652406

[19] Kutlu HR Ünsal I Görgülü M. Effects of providing dietary wood (oak) charcoal to broiler chicks and laying hens. Anim Feed Sci Technol. (2001) 90:213–26. doi: 10.1016/S0377-8401(01)00205-X

[20] Majewska T Pudyszak K Kozłowski K. The effect of charcoal addition to diets for broilers on performance and carcass parameters. Vet Med Zoot. (2011) 55:10–2.

[21] Sivilai B Preston T Leng R Hang DT Linh NQ. Rice distillers’ byproduct and biochar as additives to a forage-based diet for growing moo lath pigs; effects on growth and feed conversion. Livest Res Rural Dev. (2018) 30:111.

[22] Schmidt HP Hagemann N Draper K Kammann C. The use of biochar in animal feeding. PeerJ. (2019) 7:e7373. doi: 10.7717/peerj.7373, PMID: 31396445 PMC6679646

[23] Sun T Levin BD Guzman JJ Enders A Muller DA. Rapid Electron transfer by the carbon matrix in natural pyrogenic carbon. Nat Commun. (2017) 8:14873. doi: 10.1038/ncomms14873, PMID: 28361882 PMC5380966

[24] Chen S Rotaru AE Shrestha PM Malvankar NS Liu F. Promoting interspecies Electron transfer with biochar. Sci Rep. (2014) 4:5019. doi: 10.1038/srep05019, PMID: 24846283 PMC4028902

[25] Joseph S Kammann CI Shepherd JG Conte P Schmidt HP Hagemann N. Microstructural and associated chemical changes during the composting of a high temperature biochar: mechanisms for nitrate, phosphate and other nutrient retention and release. Sci Total Environ. (2018) 618:1210–23. doi: 10.1016/j.scitotenv.2017.09.200, PMID: 29126641

[26] Hagemann N Joseph S Schmidt HP Kammann CI Harter J. Organic coating on biochar explains its nutrient retention and stimulation of soil fertility. Nat Commun. (2017) 8:1089. doi: 10.1038/s41467-017-01123-0, PMID: 29057875 PMC5715018

[27] Gerlach H Gerlach A Schrodl W Schottdorf B Haufe S. Oral application of charcoal and humic acids to dairy cows influences Clostridium Botulinum blood serum antibody level and glyphosate excretion in urine. J Clin Toxicol. (2014) 4:186. doi: 10.4172/2161-0495.1000186

[28] Watarai S. Tana, Koiwa M. Feeding activated charcoal from bark containing wood vinegar liquid (Nekka-Rich) is effective as treatment for cryptosporidiosis in calves. J Dairy Sci. (2008) 91:1458–63. doi: 10.3168/jds.2007-0406, PMID: 18349239

[29] Wang L Gong L Zhu L Peng C Liao J. Effects of activated charcoal-herb Extractum complex on the growth performance, immunological indices, intestinal morphology and microflora in weaning piglets. RSC Adv. (2019) 9:5948–57. doi: 10.1039/C8RA10283J, PMID: 35517287 PMC9060878

[30] Wang L Zhang Y Guo X Gong L Dong B. Beneficial alteration in growth performance, immune status, and intestinal microbiota by supplementation of activated charcoal-herb Extractum complex in broilers. Front Microbiol. (2022) 13:856634. doi: 10.3389/fmicb.2022.856634, PMID: 35495714 PMC9051449

[31] Wang L Zhu L Gong L Zhang X Wang Y. Effects of activated charcoal-herb Extractum complex on antioxidant status, lipid metabolites and safety of excess supplementation in weaned piglets. Animals. (2019) 9:1151. doi: 10.3390/ani9121151, PMID: 31847500 PMC6940724

[32] Zhang Y Lin Z Wang L Guo X Hao Z. Cooperative interaction of phenolic acids and flavonoids contained in activated charcoal with herb extracts, involving cholesterol, bile acid, and Fxr/Pxr activation in broilers fed with mycotoxin-containing diets. Antioxidants. (2022) 11:2200. doi: 10.3390/antiox11112200, PMID: 36358572 PMC9686537

[33] Vuong QV Hirun S Roach PD Bowyer MC Phillips PA. Effect of extraction conditions on Total phenolic compounds and antioxidant activities of Carica Papaya leaf aqueous extracts. J Herb Med. (2013) 3:104–11. doi: 10.1016/j.hermed.2013.04.004

[34] John B Sulaiman C George S Reddy V. Spectrophotometric estimation of total alkaloids in selected justicia species. Int J Pharm Pharm Sci. (2014) 6:647–8.

[35] Alam MA Juraimi A Rafii M Hamid A Aslani F. Effects of salinity and salinity-induced augmented bioactive compounds in purslane (Portulaca Oleracea L.) for possible economical use. Food Chem. (2015) 169:439. doi: 10.1016/j.foodchem.2014.08.019, PMID: 25236249

[36] Guo WR Ou SX Long WP Wei Z Yan X. Simultaneous detection method for mycotoxins and their metabolites in animal urine by using impurity adsorption purification followed by liquid chromatography-tandem mass detection. J Chromatogr Techn. (2015) 6:1. doi: 10.4172/2157-7064.1000308

[37] Gasparotto J Kunzler A Senger MR Souza CSF Simone SG. N-acetyl-cysteine inhibits liver oxidative stress markers in Balb/C mice infected with Leishmania Amazonensis. Mem Inst Oswaldo Cruz. (2017) 112:146–54. doi: 10.1590/0074-02760160403, PMID: 28177049 PMC5293124

[38] Alexandre S Vital ACP Mottin C Do Prado RM Ornaghi MG. Use of alginate edible coating and basil (Ocimum Spp) extracts on beef characteristics during storage. J Food Sci Technol. (2021) 58:3835–43. doi: 10.1007/s13197-020-04844-1, PMID: 34471307 PMC8357873

[39] Jing T Zhao X. The improved pyrogallol method by using terminating agent for superoxide dismutase measurement. Prog Biochem Biophys. (1995) 22:84–6.

[40] Leonardo EMC Watt MS Pearse GD Dash JP Persson HJ. Comparison of tandem-X Insar data and high-density Als for the prediction of Forest inventory attributes in plantation forests with steep terrain. Remote Sens Environ. (2020) 246:111833. doi: 10.1016/j.rse.2020.111833

[41] Habibian M Sadeghi G Karimi A. Comparative effects of powder, aqueous and methanolic extracts of purslane (Portulaca Oleracea L.) on growth performance, antioxidant status, abdominal fat deposition and plasma lipids in broiler chickens. Anim Prod Sci. (2018) 59:89–100. doi: 10.1071/AN17352

[42] Ma Y Bao Y Zhang W Ying X. And its antioxidant activities. Nat Prod Res. (2020) 34:2276–82. doi: 10.1080/14786419.2018.1534852 30580585

[43] Wang S Li J Sun J Zeng KW Cui JR. No inhibitory guaianolide-derived terpenoids from artemisia argyi. Fitoterapia. (2013) 85:169–75. doi: 10.1016/j.fitote.2012.12.005, PMID: 23262266

[44] Yao D Vlessidis AG Gou Y Zhou X Zhou Y. Chemiluminescence detection of superoxide anion release and superoxide dismutase activity: modulation effect of Pulsatilla Chinensis. Anal Bioanal Chem. (2004) 379:171–7. doi: 10.1007/s00216-004-2527-z 14985908

[45] Yu C Chen J Huang L. A study on the antitumour effect of Total flavonoids from Pteris Multifida Poir in H22 tumour-bearing mice. Afr J Tradit Complement Altern Med. (2013) 10:459–63. doi: 10.4314/ajtcam.v10i6.11, PMID: 24311869 PMC3847384

[46] Vinus RD Sheoran N Maan N Tewatia B. Potential benefits of herbal supplements in poultry feed: a review. Pharma Innov J. (2018) 7:651–6.

[47] Hanchai K Trairatapiwan T Lertpatarakomol R. Drinking water supplemented with wood vinegar on growth performance, intestinal morphology, and gut microbial of broiler chickens. Vet World. (2021) 14:92–6. doi: 10.14202/vetworld.2021.92-96 33642791 PMC7896902

[48] Ruttanavut J Yamauchi K Goto H Erikawa T. Effects of dietary bamboo charcoal powder including vinegar liquid on growth performance and histological intestinal change in Aigamo ducks. Int J Poult Sci. (2009) 8:229–36. doi: 10.3923/ijps.2009.229.236 19234936

[49] Samanya M Yamauchi K-e. Morphological changes of the intestinal villi in chickens fed the dietary charcoal powder including wood vinegar compounds. J Poult Sci. (2001) 38:289–301. doi: 10.2141/jpsa.38.289

[50] Watarai S. Tana. Eliminating the carriage of Salmonella Enterica Serovar Enteritidis in domestic fowls by feeding activated charcoal from bark containing wood vinegar liquid (Nekka-Rich). Poult Sci. (2005) 84:515–21. doi: 10.1093/ps/84.4.515, PMID: 15844805

[51] Justesen U. Negative atmospheric pressure chemical ionisation low-energy collision activation mass spectrometry for the characterisation of flavonoids in extracts of fresh herbs. J Chromatogr A. (2000) 902:369–79. doi: 10.1016/S0021-9673(00)00861-X, PMID: 11192169

[52] Shulman AI Mangelsdorf DJ. Retinoid X receptor heterodimers in the metabolic syndrome. N Engl J Med. (2005) 353:604–15. doi: 10.1056/NEJMra043590, PMID: 16093469

[53] Chen Y Tang Y Guo C Wang J Boral D. Nuclear receptors in the multidrug resistance through the regulation of drug-metabolizing enzymes and drug transporters. Biochem Pharmacol. (2012) 83:1112–26. doi: 10.1016/j.bcp.2012.01.030, PMID: 22326308 PMC3339266

[54] Dong X Tong J. Different susceptibility to fatty liver-Haemorrhagic syndrome in Young and older layers and the interaction on blood Ldl-C levels between Oestradiols and high energy-low protein diets. Br Poult Sci. (2019) 60:265–71. doi: 10.1080/00071668.2019.1571164, PMID: 30657354

[55] Nie C Wang Y Liu Y Liu J Ge W. Impacts of dietary protein from fermented cottonseed meal on lipid metabolism and Metabolomic profiling in the serum of broilers. Curr Protein Pept Sci. (2020) 21:812–20. doi: 10.2174/1389203721666200203152643, PMID: 32013830

[56] Chambers KF Day PE Aboufarrag HT Kroon PA. Polyphenol effects on cholesterol metabolism via bile acid biosynthesis, Cyp7a1: a review. Nutrients. (2019) 11:2588. doi: 10.3390/nu11112588, PMID: 31661763 PMC6893479

[57] Kutlu H. Effects of dietary wood charcoal on performance and fatness of broiler chicks. Br Poult Sci. (1998) 39:31–2. doi: 10.1080/00071669888214, PMID: 10188032

[58] Kutlu H Unsal I Gorgulu M Baykal L. Effects of providing dietary wood charcoal to broiler chicks of different ages. Br Poult Sci. (1999) 40:34–5. doi: 10.1080/00071669986710, PMID: 10661431

[59] Storz G Imlay JA. Oxidative stress. Curr Opin Microbiol. (1999) 2:188–94. doi: 10.1016/S1369-5274(99)80033-2 10322176

[60] Matés JM Pérez-Gómez C. Antioxidant enzymes and human diseases. Clin Biochem. (1999) 32:595–603. doi: 10.1016/S0009-9120(99)00075-2 10638941

[61] Jiang F Lin Y Miao L Hao J. Addition of bamboo charcoal to selenium (se)-Rich feed improves growth and antioxidant capacity of blunt snout bream (Megalobrama Amblycephala). Animals. (2021) 11:2585. doi: 10.3390/ani11092585, PMID: 34573550 PMC8465871

[62] Zhang J Hu Z Lu C Bai K Zhang L. Effect of various levels of dietary curcumin on meat quality and antioxidant profile of breast muscle in broilers. J Agric Food Chem. (2015) 63:3880–6. doi: 10.1021/jf505889b, PMID: 25823972

[63] Falkoff RJ Muraguchi A Hong JX Butler JL Dinarello CA. The effects of interleukin 1 on human B cell activation and proliferation. J Immunol. (1983) 131:801–5. doi: 10.4049/jimmunol.131.2.801, PMID: 6602846

[64] Harris DP Goodrich S Gerth AJ Peng SL. Regulation of Ifn-gamma production by B effector 1 cells: essential roles for T-bet and the Ifn-gamma receptor. J Immunol. (2005) 174:6781–90. doi: 10.4049/jimmunol.174.11.6781, PMID: 15905519

[65] Dinarello CA. Immunological and inflammatory functions of the Interleukin-1 family. Annu Rev Immunol. (2009) 27:519–50. doi: 10.1146/annurev.immunol.021908.132612 19302047

[66] Bhattacharyya A Chattopadhyay R Mitra S Crowe SE. Oxidative stress: an essential factor in the pathogenesis of gastrointestinal mucosal diseases. Physiol Rev. (2014) 94:329–54. doi: 10.1152/physrev.00040.2012, PMID: 24692350 PMC4044300

[67] Singh U Devaraj S. Oxidative stress, and inflammation. Annu Rev Nutr. (2005) 25:151–74. doi: 10.1146/annurev.nutr.24.012003.132446 16011463

[68] Panneerselvam D, Statpearls, StatPearls Publishing (2023) 32491389

[69] Willenborg S Lucas T van Loo G Knipper JA Krieg T. Ccr2 recruits an inflammatory macrophage subpopulation critical for angiogenesis in tissue repair. Blood. (2012) 120:613–25. doi: 10.1182/blood-2012-01-403386, PMID: 22577176

[70] Chujo S Shirasaki F Kondo-Miyazaki M Ikawa Y Takehara K. Role of connective tissue growth factor and its interaction with basic fibroblast growth factor and macrophage chemoattractant Protein-1 in skin fibrosis. J Cell Physiol. (2009) 220:189–95. doi: 10.1002/jcp.21750, PMID: 19277979

[71] Nagao Y Sata M. Serum albumin and mortality risk in a Hyperendemic area of Hcv infection in Japan. Virol J. (2010) 7:375. doi: 10.1186/1743-422X-7-375, PMID: 21194423 PMC3022684

[72] Tuczek H-V Fritz P Grau A Mischlinsky A Wagner T. Distribution of albumin in Normal and regenerating livers of mice. Histochem. (1985) 83:165–9. doi: 10.1007/BF00495148, PMID: 3900014

[73] Tang SGH Sieo CC Ramasamy K Saad WZ Wong HK. Performance, biochemical and haematological responses, and relative organ weights of laying hens fed diets supplemented with prebiotic, probiotic and synbiotic. BMC Vet Res. (2017) 13:1–12. doi: 10.1186/s12917-017-1160-y, PMID: 28814309 PMC5559823

